# Monitoring of Rivaroxaban Therapy in Hypercoagulable Dogs

**DOI:** 10.1111/jvim.70014

**Published:** 2025-02-19

**Authors:** Erin M. Phillips, Shauna L. Blois, Anthony C. G. Abrams‐Ogg, R. Darren Wood, Gabrielle Monteith, Benoît Cuq

**Affiliations:** ^1^ Department of Clinical Studies, Ontario Veterinary College University of Guelph Guelph Ontario Canada; ^2^ Department of Pathobiology, Ontario Veterinary College University of Guelph Guelph Ontario Canada; ^3^ Department of Small Animal Clinical Sciences, School of Veterinary Medicine University College Dublin Dublin Ireland

**Keywords:** anti‐Xa assay, prothrombin time (PT), thrombin generation

## Abstract

**Background:**

Measurement of rivaroxaban efficacy using the rivaroxaban‐specific anti‐Xa assay (raXa) can be used for monitoring in veterinary medicine. Detection of rivaroxaban efficacy using other hemostatic tests would make monitoring timelier and more accessible.

**Objectives:**

Compare results of raXa with prothrombin time (PT), activated partial thromboplastin time (aPTT), fibrinogen concentration, tissue factor (TF) and kaolin‐activated thromboelastography (TEG), and thrombin generation (TG) in hypercoagulable dogs.

**Animals:**

Twelve client‐owned dogs, diagnosed with hypercoagulability or thromboembolic disease, and prescribed rivaroxaban, were recruited from a tertiary referral hospital from 2020 to 2022.

**Methods:**

Prospective clinical trial. Jugular vein blood samples were collected before treatment, and 1 week and 1–3 months after initiation of rivaroxaban therapy. Hemostatic tests were performed at each visit (3 h after rivaroxaban dosing). TG curve parameters lag time, endogenous thrombin potential (ETP), peak, and time to peak (ttpeak) were assessed.

**Results:**

There was a significant linear relationship between raXa and PT (*r* ^2^ = 0.74, *p* < 0.001), ETP (*r* ^2^ = 0.83, *p* < 0.001), lag time (*r* ^2^ = 0.87, *p* < 0.001), peak (*r* ^2^ = 0.86, *p* < 0.001), and ttpeak (*r* ^2^ = 0.86, *p* < 0.001). There was a weak linear relationship between raXa and kaolin‐activated TEG parameter reaction time (*R*) (*r* ^2^ = 0.49, *p* = 0.026). There was no significant relationship between raXa and aPTT, fibrinogen concentration and the remainder of the TEG variables (*p* > 0.05).

**Conclusion and Clinical Importance:**

PT and TG correlated with raXa. PT performed at a reference laboratory appeared to be a convenient method to monitor a small cohort of dogs receiving rivaroxaban therapy.

**Abbreviations**
aPTT: activated partial thromboplastin time
AUC: area under the curve
CAT: calibrated automated thrombogram
CI: confidence interval
CURATIVE: Consensus on the Rational Use of Antithrombotics in Veterinary Critical Care)
DOAC: direct oral anti‐coagulant
ETP: endogenous thrombin potential
GIST: gastrointestinal stromal tumor
HAC: hyperadrenocorticism
IMHA: immune‐mediated hemolytic anemia
*K*
: clotting time
MA: maximal amplitude
PLE: enteropathies
PLN: protein‐losing nephropathies
PPP: platelet‐poor plasma
PT: prothrombin time
*R*
: reaction time
raXa: rivaroxaban specific anti‐factor Xa assay
RI: reference interval
sens: sensitivity
spec: specificity
T0: baseline
T1: 1–2 weeks after starting treatment
T2: 1–3 months of treatment
TEG: thromboelastography
TEGK: kaolin activated thromboelastography
TEGTF: tissue factor activated thromboelastography
TF: tissue factor
TG: thrombin generation
ttpeak: time to peak

## 1 Introduction

Hypercoagulability, thrombosis, and the use of antithrombotic drugs are important areas of research in veterinary medicine, with thrombosis being an important cause of morbidity and death in companion animals [1, 2, 3, 4, 5, 6, 7, 8, 9, 10, 11, 12, 13]. The 2019 and 2022 Consensus on the Rational Use of Antithrombotics in Veterinary Critical Care (CURATIVE) guidelines recommend the routine use of antithrombotics in dogs at high risk for thromboembolism, such as those with heartworm disease, immune‐mediated hemolytic anemia (IMHA), severe or necrotizing pancreatitis, protein‐losing nephropathy (PLN), or protein‐losing enteropathy (PLE), or in dogs with more than one risk factor [6, 8].

Rivaroxaban is a direct oral anticoagulant (DOAC) that has been increasingly used for the prevention and treatment of thrombosis in dogs and cats [14, 15, 16, 17, 18]. Rivaroxaban inhibits factor Xa, which plays a critical role in thrombin generation. In people, its oral formulation and reduced need for close hemostatic monitoring make it a favorable alternative to traditionally used antithrombotics (e.g., heparin or warfarin). These factors have also made rivaroxaban an appealing antithrombotic for use in veterinary medicine.

The rivaroxaban specific anti‐Xa assay (raXa) is recommended for monitoring rivaroxaban in humans due to its strong correlation with plasma drug concentrations and low intra‐ and inter‐ assay variability [19]. In studies of healthy dogs, rivaroxaban caused a dose‐dependent change in prothrombin time (PT), activated partial thromboplastin time (aPTT), raXa, thrombin generation (TG) [14], and *R* value of thromboelastography (TEG) using strong activators [17, 18]. A previous pharmacodynamic study recommended monitoring coagulation in healthy dogs receiving rivaroxaban by measuring raXa or TG [17].

The use of raXa and TG assays for routine monitoring is not widely accessible in a timely manner, as they are only performed at certain reference or research laboratories. The PT is a relatively inexpensive and more widely available hemostatic test with the potential to receive same‐day results. This could make PT a more ideal monitoring test for rivaroxaban in comparison to raXa and TG assays. Two studies of healthy dogs demonstrated a strong correlation between PT and raXa in comparison to other hemostatic tests [18, 20]. However, these studies mainly evaluated healthy dogs at a single time point.

Reports describing the use of rivaroxaban in clinically ill dogs use variable doses and often do not describe hemostatic evaluation of dogs [16, 21, 22, 23, 24, 25, 26, 27, 28]. Typically in these reports, the rivaroxaban doses used were lower than those recommended in the CURATIVE guidelines (1–2 mg/kg/day) [29]. Studies evaluating the recommended rivaroxaban dose with hemostatic monitoring in ill dogs are lacking. The use of PT to monitor rivaroxaban‐treated hypercoagulable dogs requires further investigation.

The first objective of this study was to compare the results of raXa with PT, aPTT, fibrinogen concentration, TF and kaolin‐activated TEG, and TG activity in dogs with hypercoagulability and/or thrombosis treated with rivaroxaban. The second objective was to describe the clinical features of a cohort of dogs with thrombosis that were prescribed rivaroxaban for the prevention or treatment of thromboembolic disease. We hypothesized that the dose of rivaroxaban would correlate with raXa activity, and that PT would have a strong correlation with raXa activity, in hypercoagulable dogs.

## 2 Materials and Methods

### 2.1 Study Cohort

Client‐owned dogs diagnosed with hypercoagulability and prescribed rivaroxaban were recruited from a tertiary referral hospital from August 2020 to September 2022. Inclusion criteria were a diagnosis of hypercoagulability, which was made based on one or more findings: (1) ultrasound or CT imaging findings consistent with thrombosis; (2) laboratory evidence of hypercoagulability based on a kaolin‐activated TEG *G* value above the institutional reference interval; or (3) clinical signs highly suspicious of thrombotic disease. Exclusion criteria were dogs with hematocrit < 0.30 L/L and/or those receiving additional anticoagulant therapy. Concurrent antiplatelet therapy with clopidogrel was permitted. All dogs received a commercially available formulation of rivaroxaban (Xarelto, Janssen Pharmaceuticals, Titusville, NJ). Rivaroxaban dosage was left to the discretion of the primary clinician.

Statistical consultation was obtained during the study design. A study describing the effects of rivaroxaban on hemostatic testing of healthy dogs was reviewed during sample size calculation [17]. Using data from that report, it was estimated that five to six dogs would be necessary to evaluate the correlation of aXa and PT. However, given that the clinically ill dogs would be a more heterogeneous population, it was recommended to double this number (10–12 dogs) to achieve adequate power of 80% or higher for investigating the sensitivity of these hemostatic tests for a pilot trial.

Client consent was obtained before the recruitment of dogs into the study. The Institutional Animal Care Committee approved this research, and established standards were followed during this study.

### 2.2 Blood Collection and Storage

Blood samples were collected 3 h after rivaroxaban administration, at baseline (T0), 1–2 weeks after starting treatment (T1), and after 1–3 months of treatment (T2). These timeframes were chosen to assess for any cumulative rivaroxaban effect over time. At each recheck a history was taken, specifically inquiring about adverse effects and drug tolerance since the last visit. Dogs were assessed by a clinician to re‐evaluate for clinical signs associated with thrombosis (if originally present). Thrombi that could be visualized via ultrasound examination were monitored at 4 and 8 weeks during the study period, often concomitantly with the dog's T2 recheck, to assess for response to treatment. Response to treatment was determined by improvement or resolution of thrombus on ultrasound examination, or if re‐imaging was not performed, improvement in clinical signs associated with thrombosis.

Samples were obtained by direct jugular venipuncture into six 3.2% sodium citrate evacuated tubes (1.8 mL, 9:1 blood:citrate ratio, BD Vacutainer, Beckton Dickson, Franklin Lakes, New Jersey, USA). The citrated tubes were inverted 10 times to ensure adequate mixing of citrate and blood. Immediately after collection, four vials were centrifuged (3200 *g* for 15 min) to retrieve platelet‐poor plasma (PPP). The PPP was divided into 0.5–1.0 mL aliquots and stored at −80°C until TG was batch analyzed. The remaining two vials were used for TEG, raXa, PT, aPTT, and fibrinogen analysis.

### 2.3 Hemostatic Testing

At each timepoint (T0, T1, T2) PT, aPTT, fibrinogen concentration, kaolin, and TF‐activated TEG, TG, and raXa concentration were measured.

The PT, aPTT, and fibrinogen concentration were analyzed within 12 h of collection at a local reference lab (Animal Health Laboratory, University of Guelph, Guelph, Ontario) with citrated plasma using commercial reagents in a mechanical clot detection instrument (STA‐Compact, Diagnostica Stago, Parsippany, NJ). The STA‐Neoplastine CL Plus reagent was used for PT.

Both kaolin and TF‐activated TEG (TEG 500 Hemostasis Analyzer, Haemonetics Corp, Braintree, MA) were performed on citrated whole blood after a 30‐min rest period at room temperature. The TEGs were run simultaneously by a single operator (E.M.P.) using a previously described procedure [14, 18]. For the kaolin‐activated TEG, 1 mL of citrated whole blood was mixed with 40 μL of kaolin (Kaolin 40 μL, TEG 500 Hemostasis, Haemonetics Corp, Braintree, MA), then 340 μL of the blood: kaolin solution and 20 μL of calcium chloride (CaCl_2_ 0.2 M, TEG 500 Hemostasis, Haemonetics Corp, Braintree, MA) were added to a standard TEG pin and cup. In a separate channel, 20 μL of calcium chloride, 20 μL of TF (1:10 dilution, Dade Innovin, Siemens Healthcare Diagnostics Inc., Lieme, Germany), and 320 μL of citrated whole blood were combined to run the TF‐activated TEG (final TF dilution ratio 1:50 000). Both samples were run for 90 min at 37°C, after which the TEG variables *R*, clotting time (*K*), maximal amplitude (MA), *G*, angle, LY30, and LY60 were recorded. Only the variables *R*, *K*, MA, and *G* were chosen for analysis to facilitate comparison to similar studies assessing TEG results in dogs receiving rivaroxaban.

Plasma samples were transported overnight on ice to an outside laboratory (Comparative Coagulation Section/AHDC, Cornell University, Ithaca, NY) for measurement of raXa as previously described [15, 18, 27]. The assay is configured with a bovine‐activated factor X reagent added in excess to the test plasma and a chromogenic substrate for factor Xa. In this assay, residual, uninhibited factor Xa cleaves the chromogenic substrate such that the inverse of the color change in the reaction mixture is proportional to the drug concentration in the test plasma. Results were expressed as ng/mL for raXa, based on a calibration standard containing known rivaroxaban concentrations in human plasma that has been validated in dogs. Assay controls, consisting of rivaroxaban spiked‐human plasma, were measured before test samples to confirm assay performance.

The TG was performed using the calibrated automated thrombogram (CAT) method as previously described [30]. Before analysis, frozen citrated plasma samples were thawed in a 37°C water bath for 10 min, then centrifuged at 1000 Hz for 5 s. Plasma samples were analyzed within 30 min of thawing. The TG was measured on the Thrombinoscope (CAT Thrombinoscope, Diagnostic Stago, Asniere sur Seine, France) using software connected to an automated fluorometric microplate reader (Fluoroskan Ascent; Thermo Scientific, Waltham, MA, USA). Assays were performed using a 96‐well microplate (Immulon 2 HB; Thermo Scientific, Waltham, MA, USA). Distilled water was placed in the first row of each run as a negative control. A positive control was run once per plate using plasma from a healthy normal dog (collected at the start of the trial and stored at −80°C until use). The PPP‐Reagent (PPP Reagent 5 pM; Thrombinoscope, Diagnostic Stago, Asniere sur Seine, France) and a thrombin calibrator (Thrombin calibrator TA 20.0; Thrombinoscope, Diagnostic Stago, Asniere sur Seine, France) were added to wells at 20 μL, and plasma added at 80 μL. A buffer solution containing calcium chloride and a fluorogenic substrate (FluCa‐Kit; Thrombinoscope, Diagnostic Stago, Asnières‐sur‐Seine, France) were added to each well to initiate thrombin generation. Each sample was measured in triplicate. The thrombin calibrator and fluorescence in each plasma sample was measured at 10‐s intervals for a total of 60 min. A 10‐s interval was chosen over the standard 20 s to account for faster coagulation time in dogs compared to humans [31]. Due to the shorter interval between measurements, only half the plate was used (48 wells) per run. Data parameters recorded were lag time, ETP, peak, and ttpeak.

### 2.4 Statistical Analysis

Statistical calculations were performed using the SAS system. A general linear mixed model accounting for the random effect of dog was fit to see if any hemostatic test parameters could predict the raXa result. Effects included in the model were hemostatic parameters (PT, PTT, ETP, lagtime, etc.) as well as the visit and visit interaction with hemostatic parameter. Visit was removed from the model when not significant. Data was checked for normality with a Shapiro–Wilk test and residuals were examined. The raXa data was log‐transformed to meet the assumptions of normality. The predicted equations including *p*‐value for the slope and *r* ^2^ of the model were calculated.

Statistical analysis of the change in angle, LY30 and LY60 values between the three time points was only possible for kaolin‐TEG, due to insufficient TF‐TEG samples at T0. A non‐parametric Wilcoxon signed‐rank test was used to compare T1 to T2 for angle, LY30 and LY60. To compare T2 to T0 for angle, LY30 and LY60, a paired *t*‐test was used as the differences were normally distributed.

The raXa data were turned into a binary run twice; one for above the reference interval and one for below the reference interval. Logistic regression, ROC analysis and area‐under‐the‐curve were used to identify tests able to predict raXa above or below the reference interval. Using results from the logistic regression analysis, a list of Youden values and cut points were calculated. Significance was set at *p* < 0.05. Spearman correlations between raXa concentration and other hemostatic tests, as well as raXa concentration and rivaroxaban dose, were also reported to allow for easier comparison to similar previous research. The strength of association for absolute values of *r* chosen for our study was as follows: 0–0.19 = very weak, 0.2–0.39 = weak, 0.4–0.59 = moderate, 0.6–0.79 = strong, and 0.8–1.0 = very strong.

## 3 Results

### 3.1 Study Cohort

There were 12 hypercoagulable dogs included in the study. Characteristics of the study cohort, including the reason for inclusion, rivaroxaban dose used and response to treatment, are described in Appendix S1. The study cohort had a mix of male and female dogs, with a median age of 8.3 years (2 months to 13 years), and a variety of breeds. Most dogs (11/12) had a thrombus diagnosed via imaging (ultrasound = 8, computed tomography = 1, magnetic resonance imaging = 1): the external iliac artery was the most common site of thrombosis in the study (3/12 dogs), with other sites being thrombosis of the renal vein (1/12), portal, splenic, and gastric vein (1/12), jugular vein (1/12), splenic vein (1/12), aorta (1/12), right forelimb (1/12), carotid artery (1/12), and splenic infarct (1/12). Finally, one dog was diagnosed based on neurologic clinical signs suggesting thrombosis (transient ischemic attacks) and a hypercoagulable TEG tracing. The majority of dogs (11/12) had at least one concurrent disorder identified. Pancreatitis (4/12) and PLN (3/12) were the most identified underlying pathologies to explain a hypercoagulable state. Other underlying disorders identified included a gastrointestinal stromal tumor (GIST; 1/12), suspected vaccine reaction (1/12), hypoadrenocorticism (1/12) and hyperadrenocorticism (HAC; 1/12). Only one dog did not have an identifiable concurrent condition. Serial monitoring of hemostatic testing (> 1 recheck) was performed in 11/12 dogs.

### 3.2 Rivaroxaban Dosing, raXa Results and Adverse Effects

The initial rivaroxaban dose was a mean of 1.27 mg/kg/day (0.64 mg/kg twice daily, range 0.5–2 mg/kg/day) at T1, and mean of 1.43 mg/kg/day (0.71 mg/kg twice daily) and median of 0.75 mg/kg twice daily at T2. The range was 0.5–2.0 mg/kg at once daily dosing, and 0.5–1.0 mg/kg at twice daily dosing. Two dogs received concurrent treatment with clopidogrel (one dog initiated at T2 at a dose of 1.5 mg/kg q24h, another dog initiated at T1 at a dose of 1.9 mg/kg q24h) due to clinician preference. Sixty‐seven percent of dogs had partial improvement (5/12) to complete resolution (3/12) of their thrombosis based on monitoring via ultrasound examination and/or assessment of clinical signs, at 4‐ and 8‐weeks post treatment. The remainder of dogs (4/12) were either unchanged or had progression of thrombi despite therapy.

A raXa therapeutic range of 150–250 ng/mL was chosen for our study, based on a similar range used in a previous study assessing raXa activities in dogs receiving rivaroxaban [27]. The mean raXa activity was 158 ng/mL (range 20–466 ng/mL), with 13/23 of the activities in the subtherapeutic range, 7/23 within the therapeutic range, and 3/23 above the therapeutic range. There was no correlation between rivaroxaban dose and corresponding raXa result at once daily (*p* = 0.91) or twice daily (*p* = 0.44) dosing.

Approximately two‐thirds of the cohort showed partial to complete improvement of their thrombus or clinical signs associated with hypercoagulability while receiving treatment. Adverse events were noted in one dog, which had mild hematochezia 2 weeks after starting treatment. The hematochezia resolved with supportive care and without a change in rivaroxaban dose. This dog had a subtherapeutic raXa activity at the time the adverse effect occurred.

### 3.3 Hemostatic Testing Results

The means and standard deviations of selected hemostatic tests, as well as the correlation between these tests and raXa activities for select variables, are summarized in Appendix S2.

There was a strong correlation between raXa and PT (*r* = 0.71, *p* < 0.001). The raXa was strongly to very strongly positively correlated with TG variables *ttpeak* (*r* = 0.84, *p* < 0.001) and *lag time* (*r* = 0.84, *p* < 0.001), and negatively correlated with TG variables *ETP* (*r* = −0.78, *p* < 0.001) and *peak* (*r* = −0.82, *p* < 0.001). There was a weak correlation between raXa and the *R* value of kaolin‐ activated TEG (*r* = 0.39, *p* = 0.036). There was no significant relationship between raXa and aPTT, fibrinogen concentration, and all other TEG variables (*r* < 0.40, *p* > 0.05).

The mean of the TEG variables for LY30 and LY60 were within the normal reference intervals at all time points. The standard deviations of LY30 and LY60 for TF TEG were outside the upper reference interval for T1. There was no significant difference in the kaolin‐TEG variable angle between T2 and T0 (*p* = 0.015) and T1 and T2 (*p* = 0.164). For the kaolin‐TEG variables LY30 and LY60, there was no significant difference between T2 and T0 (*p* = 0.87, *p* = 0.48) and T1 and T2 (*p* = 0.46, *p* = 0.68), respectively.

### 3.4 Results of Mixed Linear Regression Model

**FIGURE 1:**
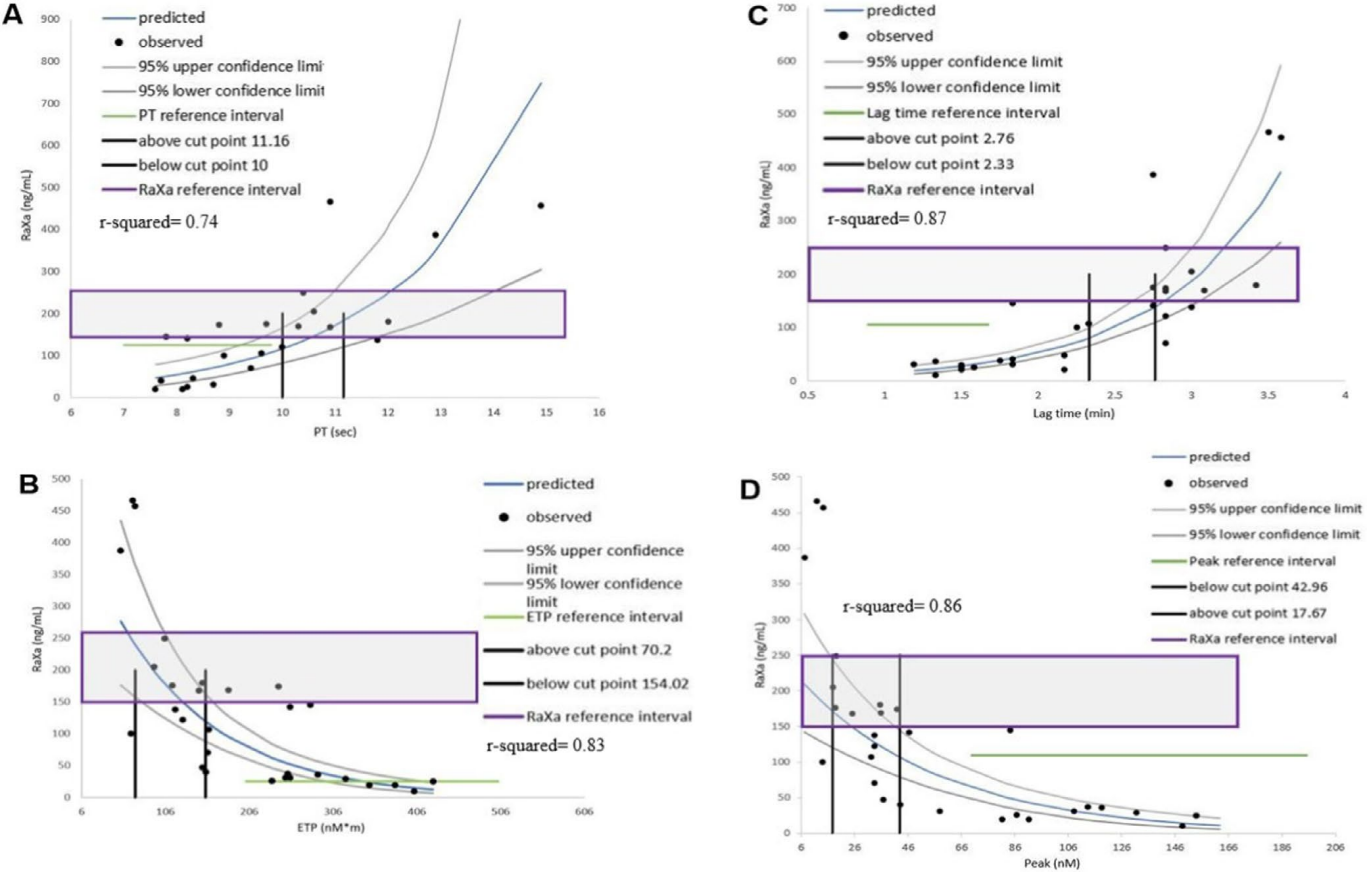
Serial hemostatic test results (PT, *ETP*, *lag time*, *peak*, raXa) in a group of 12 hypercoagulable dogs treated with rivaroxaban. Linear regression model shows a significant linear relationship between raXa and (A) PT (linear slope = 0.3968, *r* ^2^ = 0.74, *p* < 0.001), (B) *ETP* (linear slope = −0.8129, *r* ^2^ = 0.83, *p* < 0.001), (C) *lag time* (linear slope = 1.2689, *r* ^2^ = 0.87, *p* < 0.001, (D) *peak* (linear slope = −0.0188, *r* ^2^ = 0.86, *p* < 0.001). Predicted equations for significant predictors for the model: log raXa = 0.379 (pt) + 0.964, log raXa = −0.018 (peak) + 5.48, log raXa = 1.2689 (lagtime) + 1.42, log raXa = −0.008 (ETP) + 6.05. The blue curved line represents the mean predictive values of each test created using the model, and the black dots are the observed values of each dog for the corresponding tests. The gray curved lines represent the upper and lower confidence interval for each test, and the purple box represents the reference range for raXa. The green horizontal line represents the reference interval for each parameter, and the black vertical lines indicate the upper and lower cut points for each hemostatic test.

The graphs for raXa compared to PT and the TG parameters *ETP*, *lag time*, and *peak*, are shown in Figure 1. There was a significant linear relationship between raXa and PT (*r* ^2^ = 0.74, *p* < 0.001), *ETP* (*r* ^2^ = 0.83, *p* < 0.001), *lag time* (*r* ^2^ = 0.87, *p* < 0.001), *peak* (*r* ^2^ = 0.86, *p* < 0.001), and *ttpeak* (*r* ^2^ = 0.86, *p* < 0.001).

There was a weak linear relationship between raXa and the kaolin‐activated TEG parameter *R* (*r* ^2^ = 0.49, *p* = 0.026). There was no significant relationship between raXa and aPTT, fibrinogen concentration and the remainder of the TEG variables (*p* > 0.05).

### 3.5 Cut Points, Sensitivity and Specificity for PT and TG Parameters

The cut points below the reference intervals for selected hemostatic tests, as well as the area‐under‐the‐curve, confidence interval, *p*‐values, sensitivity and specificity, are summarized in Table 1. The lower cut point of PT (10.01 s) had the greatest sensitivity for predicting a subtherapeutic raXa level, with a sensitivity of 0.96. The lower cut points for *peak* (42.96 nM) and *ttpeak* (6.57 min) had the highest specificity (1.0) for predicting a subtherapeutic raXa activity.

**TABLE 1:** Cut points below the reference range for hemostatic tests to predict subtherapeutic raXa levels (< 150 ng/mL).

Test	Cut point	AUC	CI	*p*	Sens	Spec
PT (s)	10.01	0.93	0.85–1.0	< 0.0001	0.96	0.80
*lag time* (min)	2.33	0.93	0.85–1.0	< 0.0001	0.80	0.90
*ETP* (nM.min)	154.02	0.86	0.73–0.99	< 0.0001	0.80	0.80
*peak* (nM)	42.96	0.89	0.78–1.0	< 0.0001	0.70	1.0
*ttpeak* (min)	6.57	0.98	0.93–1.0	< 0.0001	0.95	1.0

Abbreviations: AUC, area under the curve; CI, confidence interval; Sens, sensitivity; Spec, specificity.

Table 2 summarizes similar data for cut points above the reference intervals for selected hemostatic tests. The higher cut points with the greatest sensitivity for predicting a supratherapeutic raXa activity were PT (11.16 s), *lag time* (12.68 min), *ETP* (70.20 nM.min), *peak* (17.87 nM), and *ttpeak* (7.2 min), all with a sensitivity of 1.0. The higher cut point for *lag time, ETP* and *peak* had the highest specificities (0.96) for predicting a supratherapeutic raXa activity. The sensitivity and specificity were chosen based on which value produced the highest Youden number.

**TABLE 2:** Cut points above the reference range for hemostatic tests to predict supratherapeutic raXa levels (> 250 ng/mL).

Test	Cut point	AUC	CI	*p*	Sens	Spec
PT (s)	11.16	0.97	0.92–1.0	< 0.0001	1.0	0.89
*lag time* (min)	12.68	0.87	0.62–1.0	< 0.0001	1.0	0.96
*ETP* (nM.min)	70.20	0.87	0.65–1.0	< 0.0001	1.0	0.96
*peak* (nM)	17.87	0.98	0.95–1.0	< 0.0001	1.0	0.96
*ttpeak* (min)	7.2	0.97	0.91–1.0	< 0.0001	1.0	0.88

Abbreviations: AUC, area under the curve; CI, confidence interval; Sens, sensitivity; Spec, specificity.

## 4 Discussion

This study describes a cohort of clinically ill dogs prescribed rivaroxaban after being diagnosed with thrombosis or having a presumed high risk of thrombosis. Serial monitoring was performed on these dogs using a range of hemostatic tests, with PT and TG being strongly correlated with raXa.

There was a strong correlation between raXa, PT and all TG parameters in our study. Thrombin generation variables had the best correlation with raXa activities in the present and in previous studies [17, 18]. Given that TG is only performed in research settings, this method does not provide a readily available monitoring alternative in a clinical setting. The strong correlation between PT and raXa suggests that PT could be a convenient monitoring test in the small cohort of dogs receiving rivaroxaban therapy enrolled in our study. Most of the PT measurements in our study were below or close to the normal reference interval, therefore a single measurement while on treatment could be hard to interpret unless prolongation is noted. Serial PT monitoring of dogs (ideally before and after rivaroxaban treatment) would likely provide optimal guidance for rivaroxaban dosage adjustments.

Consistent with studies in dogs with thrombosis, pancreatitis and PLN were the most identified underlying disorders in our cohort of dogs [3, 5, 7]. Dogs with PLN and those with necrotizing pancreatitis are considered at high risk of hypercoagulability and thrombosis [6]. The diagnosis of hypercoagulability in veterinary medicine is challenging. The definition as it relates to laboratory testing can be unclear and identification of hypercoagulability based on a hemostatic test might not translate to a truly increased risk of thrombosis. As suggested by Virchow's triad, hypercoagulability is only one of three possible contributing factors to thrombus development. There is no single laboratory test that can detect all abnormalities that result in a hypercoagulable state. Viscoelastic testing, such as TEG, is thought to reflect the in vivo hemostasis process more than traditional plasma‐based tests, but even these tests are unable to account for all factors that contribute to clot formation in vivo [32]. This discrepancy between TEG results and thrombosis was shown in a recent report, where dogs with confirmed thrombosis had TEGs showing a hypercoagulable or a normocoagulable pattern [33]. In our study we primarily used clinical evidence of thrombosis (i.e., thrombosis diagnosed via imaging, clinical signs highly consistent with thrombosis) to infer a hypercoagulable state, with only one dog enrolled based on TEG results in combination with clinical signs of thrombosis.

At the time of enrolment before initiation of rivaroxaban, none of the dogs were hypercoagulable based on the *G* value for TF‐activated TEG, while all dogs were hypercoagulable based on the *G* value for kaolin‐activated TEG. A similar discrepancy between kaolin versus TF‐activated TEG has been noted in cats, suggesting that kaolin could be a more potent activator than TF [34, 35]. This discrepancy could depend, at least to some extent, on the dilution of TF used, which varies between studies. One explanation for this discrepancy might be that recombinant TF is reconstituted in the laboratory before use, whereas kaolin is available pre‐made from a manufacturer. This reduces the possibility of pre‐analytical error when kaolin is used as an activator instead of TF. As TF is an initiator of coagulation in vivo, the TF‐activated TEG might be a more accurate in vitro assessment of coagulation. The use of TEG with different activators in dogs of various coagulation states requires further investigation. Rivaroxaban did not appear to have a notable influence on endogenous fibrinolytic potential, as there was a lack of significant change in angle, LY30 and LY60 on kaolin‐TEG over the three time points.

Rivaroxaban and other DOACs have predictable pharmacokinetic effects in humans and in healthy dogs [17, 36]. Routine monitoring of rivaroxaban effect is not performed in humans. However, some studies suggest that monitoring raXa activities could reduce the risk of bleeding and thrombotic events in some specific human patient populations [37, 38, 39, 40]. In these studies, target rivaroxaban‐specific aXa activities were considered the ideal way of monitoring of rivaroxaban therapy in human medicine [41, 42]. A raXa target therapeutic range of 150–250 ng/mL was chosen for our study, based on a similar range used in a previous study assessing raXa activities in dogs receiving rivaroxaban [27]. There is, however, a lack of research assessing whether this range of raXa activities accurately predicts clinical effect in dogs treated with rivaroxaban and this warrants further research.

Thromboelastography is commonly used as a global assessment of hemostasis. While the *R* value of kaolin‐activated TEG had a weak correlation with raXa activity in the present study, it was inferior to PT and TG, and the remainder of the TF and kaolin‐activated TEG variables did not correlate well with raXa activities. The utility of TEG for monitoring rivaroxaban effect was variable in previous studies of healthy dogs [17, 18, 43]. Only one study initially assessed TEG in a clinically ill dog before starting treatment with rivaroxaban, but no hemostatic monitoring (including raXa) was performed during treatment [23]. These variable findings in the ability for TEG to correlate with raXa in dogs are similar to those in humans, where the *R* value of TEG with strong activators had a strong to poor correlation with raXa activity [44, 45, 46].

The institutional reference interval for PT in the present study was 7.0–9.8 s. A PT of less than 10.01 s predicted raXa activity below the therapeutic range (< 150 ng/mL), with a sensitivity of 96% and specificity of 80%. In contrast, a PT value > 11.16 s predicted raXa activity > 250 ng/mL, with a sensitivity of 100% and specificity of 89%. A higher sensitivity was favored over specificity when choosing the cut point values, as inaccurately classifying a dog's raXa activity as sub‐ or supratherapeutic when it is truly therapeutic, was considered more deleterious than inaccurately classifying a dog's raXa activity as therapeutic. From this data, a slight elevation in PT at the study institution was considered desirable for predicting therapeutic raXa activity. Similar to a previous report, the present study found the strongest correlation between PT and raXa activity when the activity was in the subtherapeutic range (< 100 ng/mL) [18]. As more than half of the raXa activity results were considered subtherapeutic in the present study, a stronger relationship in other ranges might have been found if more data were available at raXa activities > 150 ng/mL. Due to the variability between PT assays, the utility of the cut‐off values created in our study is limited to the specific lot of PT reagents used at our reference laboratory. Further studies involving different types of PT reagents tested on a larger cohort of dogs with a greater amount of data at raXa activities > 150 ng/mL would be needed before cut‐off PT values guiding rivaroxaban therapy could be recommended.

A dose‐dependent increase in raXa activity has been previously demonstrated in healthy dogs and cats [14, 15, 17]. Similar to a previous report in ill dogs, however, no correlation was found between raXa activity and rivaroxaban dose in the present study [27]. This variable effect of rivaroxaban on raXa activity amongst a cohort of ill dogs suggests that the pharmacokinetics of rivaroxaban is less predictable in clinical illness resulting in a greater need for hemostatic monitoring during rivaroxaban treatment in such dogs.

Most of the rivaroxaban doses used in this study were within the recommended range for dogs of 1–2 mg/kg/day and typically the dose was divided into dosing every 12 h [29]. The dose of rivaroxaban tended to increase over the study timeline, based on the dog's condition and raXa activity results at rechecks. Research in healthy dogs shows that twice daily administration (8 h apart) extends the duration of rivaroxaban effect without increasing the magnitude of the anticoagulant effect [17]. Twice daily dosing led to less fluctuation in plasma rivaroxaban concentrations, compared to once daily dosing in humans [47]. Most dogs in the present study (8/12) had partial improvement to complete resolution of their thrombus by 8 weeks post‐treatment and hemorrhagic adverse effects were rare with the dosages used. Based on our study results, rivaroxaban prescribed at twice daily dosing in the range recommended by the CURATIVE guidelines (1–2 mg/kg/day) [29] was well tolerated by dogs with evidence of thrombosis and/or a hypercoagulable state, that did not have evidence of renal or hepatic insufficiency.

Despite the use of rivaroxaban within the recommended dosing range in our study, only 30% of dogs achieved therapeutic raXa activity, with most of the remaining dogs having subtherapeutic activities. Regardless, approximately two‐thirds of the cohort of dogs showed partial to complete improvement of their thrombus or clinical signs associated with hypercoagulability. This discrepancy between the raXa activity and clinical efficacy was also noted in a retrospective study where 67% of dogs had partial to complete thrombus resolution after 4 weeks of therapy despite similar raXa activities to non‐responders [27].

A possible explanation for the improvement of thromboembolic disease despite subtherapeutic raXa activities is the timing of blood monitoring in comparison to thrombus re‐evaluation. In our study, all dogs had raXa activities assessed before their first thrombus re‐evaluation. A substantial portion of dogs (67%) had their rivaroxaban doses adjusted based on their raXa activity, therefore an early dose adjustment could increase the chance of seeing a positive therapeutic result at a later recheck. Additionally, clopidogrel was prescribed concurrently in 17% of dogs with confirmed thrombosis in the present study and 82% of dogs in a previous study [27]. Though platelet function testing was not performed to confirm clopidogrel effect in either study, it is possible that treatment with clopidogrel was responsible for the efficacy of antithrombotic treatment rather than rivaroxaban. It is also possible that in some cases the initial mechanisms contributing to thrombus formation, such as endothelial injury or vascular turbulence, had resolved, and the improvement in thromboembolic disease was unrelated to drug effect.

There was a low incidence of adverse effects in the present study, which is similar to the findings in other studies [16, 17, 27, 43]. This could be due to the early monitoring performed in the study at 1–2 weeks after starting treatment, as a dosage decrease was implemented if supratherapeutic raXa activities were found. Additionally, if low raXa activities were detected, the dose of rivaroxaban was increased, likely reducing the risk of thrombus progression. In some cases, the minimal adverse effects related to thrombus progression could be due to resolution of the underlying prothrombotic condition rather than rivaroxaban treatment. Due to the poor correlation between rivaroxaban dose and raXa activity, monitoring could be desirable to ensure the prescribed dose achieves raXa activities within the therapeutic range.

There were several limitations in this study. A small cohort of dogs was recruited for our study. Though this was determined to be a sufficient sample size based on our power calculation, due to the inherent variability between dogs a larger sample size would have been ideal. Dogs were permitted to have concurrent clopidogrel therapy to aid in recruitment, but a more standardized cohort would have been ideal. When monitoring thrombus size via ultrasound examination, various radiologists performed the examinations and were not blinded, introducing possible inter‐operator variability in clot detection and measurement. The dosage of rivaroxaban was at the discretion of the clinician, and both the dose and frequency of rivaroxaban increased throughout the study. The use of a standard rivaroxaban dose throughout might have identified a dosage that more consistently resulted in a raXa activity within the therapeutic range.

A limitation of providing specific cut‐point values for PT is that they are only applicable to PT testing performed using a similar methodology. Previous studies have found the sensitivity of PT to raXa activity was highly dependent on the reagent used, with the STA‐Neoplastine being the recommended reagent to monitor rivaroxaban [48, 49]. Additionally, there were only two data points where PT was > 11.16 s and raXa was > 250 ng/mL, limiting the validity of the upper cut‐point recommendation. Due to these limitations, clinicians should be cautioned when using our PT cut points to monitor dogs receiving rivaroxaban. Future studies assessing the relationship between raXa and different PT tests (e.g., commercial laboratory, dog side testing) would be beneficial.

In conclusion, rivaroxaban appeared to be well‐tolerated by a group of hypercoagulable dogs. Most dogs in the present study had partial to complete improvement of clinical signs with minimal adverse effects. PT appeared to be a convenient method to monitor our small cohort of hypercoagulable dogs receiving rivaroxaban.

## Disclosure

Authors declare no off‐label use of antimicrobials.

## Ethics Statement

This study was approved by the University of Guelph Institutional Animal Care and Use Committee (IACUC). The authors declare human ethics approval was not needed.

## Conflicts of Interest

The authors declare no conflicts of interest.

## Supporting information


**Data S1** Supporting Information.
