# Supplementary material for: Monitoring of Rivaroxaban Therapy in Hypercoagulable Dogs
Source: J Vet Intern Med. 2025 Feb 19;39(2):e70014. doi: 10.1111/jvim.70014 (PMC11836667; doi:10.1111/jvim.70014)
Supplement: Supplementary file 1 — Data S1 Supporting Information. [file JVIM-39-e70014-s001.docx]

Appendix A: Characteristics of the study population, rivaroxaban dose, response to treatment and adverse reactions

| Patient | Signalment | Weight | Evidence of hyper-  coagulability | Underlying disorder | Rivaroxaban dose used | Response to treatment | raXa (R.I. 150-250 ng/mL) | Adverse reactions | Concurrent antithrombotics |
| --- | --- | --- | --- | --- | --- | --- | --- | --- | --- |
| Dog 1 | 9 yr FS mixed breed | 7.1 kg | Renal vein thrombus | GIST | T1= 2 mg/kg q24h  T2= 2 mg/kg  q24h | Resolved at 4-week ultrasound | T1= 145  T2= 47 | None | None |
| Dog 2 | 10 yr FS mixed breed | 23.3 kg | External iliac arterial thrombus | None identified | T1= 0.5  mg/kg q24h T2= 1.0  mg/kg q24h | Minimal-mild improvement at 4- and 8-week ultrasound | T1= 107  T2= 168 | None | None |
| Dog 3 | 11 yr MN Coonhound | 42.0 kg | External iliac arterial thrombus | PLN | T1= 0.75  mg/kg q24h T2= 0.5  mg/kg q12h | Unchanged at 4 weeks, progressive at 8-week ultrasound | T1= 122  T2= 138 | None | None |
| Dog 4 | 10 yr FS Labrador Retriever | 35.0 kg | Portal vein, splenic and gastric vein  thrombi | PLN | T2= 0.5  mg/kg q12h | Progressive on CT scan | T2= 40 | None | None |
| Dog 5 | 9 yr MI German Shepherd Dog | 44.1 kg | Seizures, suspect transient ischemic attacks | Pancreatitis | T1= 0.9  mg/kg q12h T2= 0.9  mg/kg q12h | No further episodes after treatment started | T1= 169  T2= 249 | None | None |
| Dog 6 | 6 yr FS Standard Poodle | 18.4 kg | Splenic infarct | Necrotizing pancreatitis | T1= 1.0  mg/kg q12h T2= 0.5  mg/kg q12h | Unchanged at 4- and 8- week ultrasound | T1= 387  T2= 100 | None | None |
| Dog 7 | 2 month MI Irish Wolfhound | 9.8 kg | Ischemic myelopathy,  jugular thrombus | Suspected vaccine reaction | T1= 0.5  mg/kg q12h T2= 1.0  mg/kg q12h | Partial improvement  at 4 and 8 weeks | T1= 26  T2= 70 | None | None |
| Dog 8 | 8 yr MN Dachshund | 4.2 kg | External iliac arterial thrombus | Pancreatitis | T1= 1.2  mg/kg q24h T2= 0.6  mg/kg q12h | Partial improvement at 4 weeks, resolved at 8-week ultrasound | T1= 466  T2= 205 | None | None |
| Dog 9 | 11 yr MN King Charles Cavalier Spaniel | 12.4 kg | Aortic thrombus partially occluding the renal arteries | Pancreatitis, diabetes mellitus | T1= 0.4  mg/kg q12h T2= 0.8  mg/kg q12h | Recheck ultrasound after 6 days unchanged, patient euthanized before 4-  week ultrasound | T1= 180  T2= 457 | None | Clopidogrel- T2= 1.5  mg/kg q24h |
| Dog 10 | 7 yr MN Labrador Retriever | 41.5 kg | Splenic vein thrombus | Hypoadreno-corticism | T1= 0.7  mg/kg q12h T2= 0.87  mg/kg q12h | Resolved at 4-week ultrasound | T1= 20  T2= 176 | None | None |
| Dog 11 | 6 yr FS Boston Terrier | 12.7  kg | Right forelimb thrombus | HAC | T1= 0.9  mg/kg q12h T2= 0.9  mg/kg q12h | Improved clinical signs at 4-week recheck | T1= 142  T2= 174 | None | None |
| Dog 12 | 13 yr MN Yorkshire Terrier | 4.5 kg | Carotid thrombus | PLN | T1= 0.55  mg/kg q12h T2= 1.1  mg/kg q12h | Partial improvement at 4-week ultrasound | T1= 20  T2= 31 | Transient hematochezia (2 weeks post treatment) | Clopidogrel- T1,T2= 1.9  mg/kg q24h |

*Legend: FS= female spayed, MI= male intact, MN= male neutered, PLN= protein losing nephropathy, GIST= gastrointestinal stromal cell tumor, HAC= hyperadrenocorticism, T0=baseline, T1=1^st^ recheck at 1-2 weeks, T2= 2^nd^ recheck at 1-3 months.*

Appendix B: Hemostatic data in hypercoagulable dogs treated with rivaroxaban at 3 time points

| **Test/R.I.** | **T0 (n=9) M ± SD** | **T1 (n=12) M ± SD** | **T2 (=12) M ± SD** | **P= (T0, T1,**  **T2) vs raXa** | **r= (T0, T1,**  **T2) vs raXa** |
| --- | --- | --- | --- | --- | --- |
| PT (sec)  R.I. 7.0-9.8 | 8.19 ± 0.86 | 9.6 ± 1.8 | 10 ± 1.9 | **<0.001** | **0.71** |
| aPTT (sec)  R.I.10.8-14.7 | 14.79 ± 6.13 | 13.52 ± 1.44 | 14.3 ± 1.36 | 0.195 | 0.25 |
| K-TEG R (min)  R.I. 0.8-3.0 | 3.34 ± 0.98 | 3.68 ± 1.25 | 4.18 ± 1.34 | 0.036 | 0.39 |
| TF-TEG R (min)  R.I. 1-3 | 0.58 ± 0.38 | 0.9 ± 0.45 | 1.2 ± 0.56 | 0.301 | 0.14 |
| K-TEG Angle (deg)  R.I. 47-74 | 75.58 ± 5.41 | 69.78 ± 10.81 | 66.45 ± 11.74 | - | - |
| TF-TEG Angle (deg)  R.I. 57-78 | 66.74 ± 10.45 | 64.11 ± 8.02 | 56.66 ± 11.17 | - | - |
| K-TEG LY30 (%)  R.I. 0-8 | 0.77 ± 1.97 | 0.15 ± 0.30 | 0.06 ± 0.16 | - | - |
| TF-TEG LY30 (%)  R.I. 0-8 | 0.14 ± 0.31 | 5.98 ± 10.03 | 2.1 ± 3.74 | - | - |
| K-TEG LY60 (%)  R.I. 0-15 | 1.68 ± 1.91 | 0.84 ± 1.07 | 0.99 ± 1.34 | - | - |
| TF-TEG LY60 (%)  R.I. 0-15 | 0.9 ± 0.84 | 9.86 ± 12.97 | 6.26 ± 7.60 | - | - |
| *ttpeak* (min)  R.I. 2-3.78 | 3.0 ± 0.32 | 49.61 ± 30.79 | 6.18 ± 1.03 | **<0.001** | **0.84** |
| *lag time* (min)  R.I. 0.89-1.67 | 1.44 ± 0.19 | 2.51 ± 0.71 | 2.64 ± 0.52 | **<0.001** | **0.84** |
| *ETP* (nM*min)  R.I. 202.91-503.76 | 327.78 ± 70.11 | 202.71 ± 106.84 | 138.71 ± 59.59 | **<0.001** | **-0.78** |
| *peak* (nM)  R.I. 69.86-195.13 | 133.81 ± 21.58 | 49.61 ± 30.79 | 29.59 ± 13.77 | **<0.001** | **-0.82** |
| raXa (ng/mL)  R.I. 150-250 | 28 ± 9.88 | 162.18 ± 144.07 | 154.58 ±  118.44 | - | 1.0 |

*Legend- T0=baseline, T1=1^st^ recheck at 1-2 weeks, T2= 2^nd^ recheck at 1-3 months. M=mean, SD= standard deviation. R.I.= reference interval, K-TEG= kaolin activated thromboelastography, TF-TEG= tissue factor activated thromboelastography. Bolded values= significant correlation between the hemostatic tests and raXa.*
